# Morbidity Experiences and Disability Among Canadian Women

**DOI:** 10.1186/1472-6874-4-S1-S10

**Published:** 2004-08-25

**Authors:** Marie DesMeules, Linda Turner, Robert Cho

**Affiliations:** 1 Centre for Chronic Disease Prevention and Control, Health Canada, 120 Colonnade Rd, Ottawa, Canada; 2 Canadian Institute for Health Information, Toronto, Canada; 3 Centre for Chronic Disease Prevention and Control, Health Canada, 120 Colonnade Rd, Ottawa, Canada

## Abstract

**Health Issue:**

Women are more frequently affected by chronic conditions and disability than men. Although some of these sex differences have been in part attributed to biological susceptibility, social determinants of health and other factors, these gaps have not been fully explained in the current literature. This chapter presents comparisons of hospitalization rates, and the prevalence of chronic conditions and physical disability between Canadian women and men and between various subgroups of women, adjusting for selected risk factors. The Canadian Hospital Morbidity Database (2000–2001) and Canadian Community Health Survey (2000–2001) were used to examine inpatient hospital morbidity, prevalence of chronic conditions and disability.

**Key Findings:**

Hospitalization rates were 20% higher among women than men. This was due to the large number of hospitalizations for pregnancies and childbirth. When "normal" deliveries were excluded, hospitalization rates remained higher among women. Women had slightly lower rates of hospitalizations for ambulatory-care sensitive conditions than men. Prevalence of activity limitation (mild and severe) was higher among women than men, and differences remained after adjusting for age, chronic conditions, socio-economic status, and smoking. Women who reported a disability were less likely than men to be in a partnered relationship, have less tangible social support, and have lower income and employment rates.

**Data Gaps and Recommendations:**

The impact of morbidity and disability on Canadian women is substantial. These results identify areas for interventions among more vulnerable subgroups, and point to the need for further research in the area of risk factors for the prevention of morbidity and disability in the population.

## Background

Overall morbidity is commonly defined as "departure from an overall state of health," but often refers more specifically to the effect of illness, disease or injury in a population. Although this concept is relatively narrow in the context of a broad population health assessment framework, it is nevertheless an essential component to consider when describing the health of a population.

It is well known that women have a longer life expectancy than men, and, as described in the chapter entitled "The Mortality, Life and Health Expectancy of Canadian Women," this is mainly due to a lower rate of preventable mortality among women. Women do not appear to have a similar advantage, however, when morbidity is examined, defined in a variety of ways (such as hospitalization rates, prevalence of chronic conditions and overall disability). [1,2] Canadian data from health surveys and hospital morbidity support these findings. [3] No single explanation fully accounts for these sex discrepancies, which should, rather, be examined in the context of biological, social, economic and environmental determinants.

Previous findings in Canada have shown that, although there has been a steady decline in hospitalization rates over the last several decades among women and men for most causes (though less pronounced when same-day procedures are included), women are hospitalized more frequently (especially in the younger adult years), the sex ratios being approximately 1:4 over all age groups. These findings can be explained, at least in part, by pregnancy-related hospitalizations. [4-10] However, over the 1990s, the decline in hospital services is less pronounced when same-day procedures are included. Data on visits to emergency departments show similar patterns by sex. [11,12]

The prevalence of chronic conditions, another key measure of long-term morbidity, is also generally higher among women. Previous findings indicate that women show a higher prevalence of chronic conditions such as allergies, arthritis/rheumatism, asthma, high blood pressure, and bronchitis or emphysema, which are in general not fatal but can lead to dependence and/or disability. Women have similar or slightly lower rates than men for life-threatening chronic conditions, such as coronary heart disease and cancer. [1,3,7,13-16]

The higher prevalence of disability, another key measure of population health, among women as compared with men is also fairly well established, [3,9,17-19] and is at least in part accounted for by the higher prevalence of disabling chronic conditions, such as arthritis, among women. [3,20] Various measures of disability, such as activity limitation and dependence on others for daily tasks, and composite measures, such as the Health Utility Index, [21-23] have been used to compare the functional status of various populations.

The 2001 Participation and Activity Limitation Survey (PALS), a large national survey, provides information on adults and children who indicated in the 2001 census in Canada that they had disabilities. Data from this recent survey indicate that 13.3% of women and 11.5% of men (all ages combined) report a disability. [24] However, the higher prevalence of reported disability among women is observed only in those aged 25 and over. Disability is reported more often among boys younger than 14 than among girls of the same age. [2] Important patterns by sex include the higher proportion of severe disability among women, and the corresponding higher proportion of mild disability among men. Moreover, women report a higher proportion of disability due to chronic pain and problems with mobility, while men aged 65 and over report a higher frequency of disability due to memory and hearing problems. [24]

Approximately 1% of all Canadians live in long-term care facilities, and a large proportion of them have a disability of dependency, as indicated by data from the institutional component of the National Population Health Survey (NPHS). [25]

Important patterns over the last decades have been observed. In particular, the prevalence of many chronic conditions has decreased (with the exception of diabetes and asthma) among Canadian women and men under 65 years of age over the last 20 years, [17,19,22] and the prevalence of disability has decreased among men, but not women, younger than 65. [17,19,22] The total number of Canadian women and men with chronic conditions and/or disability is expected to continue to increase because of the aging population and the increasing survival rates for many chronic conditions. [8]

### Determinants of Morbidity and Disability

A number of factors are associated with morbidity and disability, including physical/medical conditions, health behaviours and lifestyle, demographic and economic factors such as education and income, and psychosocial and cultural determinants. [26-30] After adjustment for sex-specific diagnosis and higher mortality rates among men, hospital services were found to be comparable between men and women. [18] However, lower levels of morbidity were found among employed women as compared with homemakers, particularly among women working part time. [19] A higher prevalence of activity limitation was also found in groups with lower educational attainment and income, [10,12,17,30,31] although these differences were weaker for education. Underlying conditions most often associated with activity limitations are arthritis or rheumatism, back or spine problems, and heart disease. [32,33]

Given that the prevalence and patterns of morbidity and disability among Canadian women and men have been fairly well described in the existing literature, the objectives of this chapter are to provide further insight into the factors explaining the sex and gender gap in overall morbidity experiences and disability. In addition, the social and economic profiles of the lives of women and men with disabilities are examined. Patterns of morbidity and disability among subgroups of women, particularly more vulnerable women, are described. This section examines morbidity and disability overall. Other chapters of the report present more specific information on hospital services, and disability and specific chronic conditions are also discussed in more detail in the two chapters "The Impact of Arthritis on the Women of Canada" and "Dementia/Alzheimer's Disease."

## Methods

In an attempt to measure overall morbidity, three types of indicators have been selected: hospital morbidity (separations and length of stay), prevalence of chronic conditions and disability. Data from the Canadian Community Health Survey (CCHS) (2000–2001) and only the inpatient data from acute care hospitals contained in the Hospital Morbidity Database were used (i.e. same-day procedures were excluded) for the analysis in this chapter. Disability was measured in four different ways: activity limitation, dependence on others for daily tasks, the Health Utility Index (a score less than 0.830), [21] and disability days.

The Health Utility Index is a measure of health-related quality of life, which is a broader concept, incorporating not only physical mobility but also other components of well-being such as emotion and pain. Data from the institutional components of the NPHS were also used to complement information on household respondents.

Hospitalization rates were analyzed using a number of approaches. All-cause hospitalization rates were calculated for women, as well as all causes minus all pregnancy and childbirth, and all causes minus "normal" pregnancy and childbirth. To estimate the number of normal deliveries without complications, deliveries with a most responsible diagnosis of ICD-9 code 650 were identified. Hospitalization rates for "ambulatory care sensitive conditions," or conditions in which appropriate ambulatory care prevents or reduces the need for admission to hospital, were analyzed.

In the CCHS, individuals were considered to have a long-term activity limitation if they answered "yes" to the question about whether they were limited at home, at school, at work or in other situations because of health problems. Long-term activity limitation was defined as limitation in the kind or amount of activity because of a long-term physical or mental condition or a health problem that had lasted or was expected to last six months or more. Chronic conditions were categorized into mild, moderate and severe according to level of impact on functional status. [21] Disability was also categorized, as moderate or severe. [24] Individuals who had activity limitation and dependency were considered to have severe disability. The distributions of socio-demographic variables among women and men who reported a disability were compared. Bivariate and multivariate (logistic regression) analysis techniques were used to identify determinants of morbidity and disability among Canadian women as compared with men using CCHS data. In the analysis a weighted approach was used to account for the complex survey design.

## Results

### Hospitalizations

**Figure 1:**
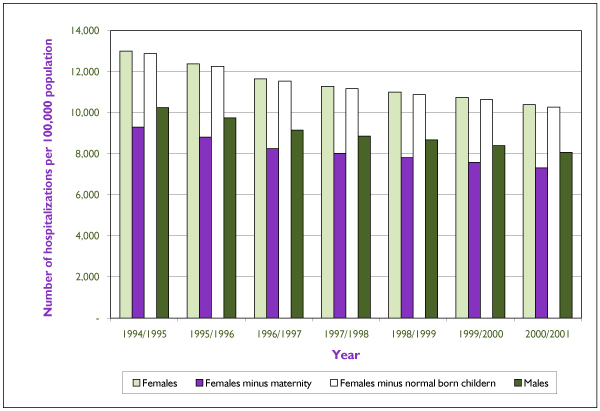
**Age-Standardized Hospitalization Rates Males and Females, 1994–1995 through 2000–2001, Canada **Source: Hospital Morbidity Database, CIHI

As shown in Figure 1, hospitalization rates among men and women have been decreasing in recent years, and hospitalization rates among women are higher than among men across all years. The decreasing rate over time remains true even when hospitalizations for pregnancy and childbirth are excluded, although the difference by sex becomes smaller.

**Figure 2:**
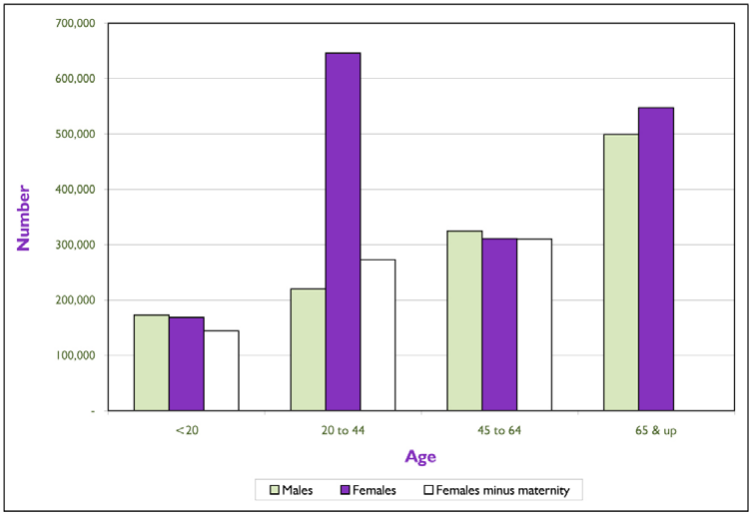
**Number of Hospitalizations in Acute Care Hospitals 2000–2001, Canada **Source: Hospital Morbidity Database, CIHI

Similarly, when numbers of inpatient hospitalizations for men and women are compared within age groups (Figure 2), there are somewhat more inpatient hospitalizations among men younger than 20 and men between 45 and 64 years of age. More women than men between the ages of 20 and 44 are hospitalized, however, whether or not hospitalizations for maternity are included. Moreover, approximately 8% to 10% of hospitalizations for deliveries involved normal deliveries without complications. When pregnancy and childbirth with complications were included in the all-cause hospitalization rates for women (providing a more accurate assessment of women's morbidity), rates were approximately 20% higher than those of men in the 20 to 44 age group (data not shown).

**Figure 3:**
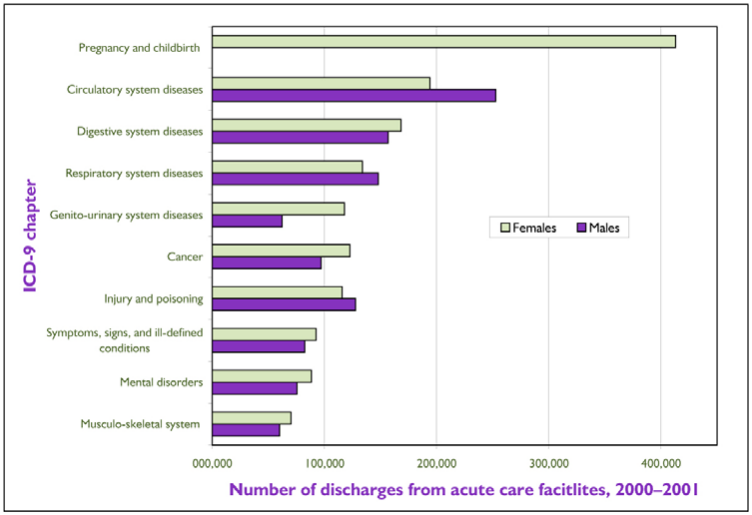
**Top Ten Reasons Why Women are Hospitalized Compared with Numbers of Hospitalizations Among Men for the Same Cause, Canada **Source: Hospital Morbidity Database, CIHI

The most responsible diagnoses for hospitalization vary between women and men, as indicated in Figure 3. For example, pregnancy and childbirth were the most common causes of hospitalization among women (approximately 25% of all hospitalizations). When causes other than pregnancy and childbirth were examined, the proportion of all hospitalizations due to injury/poisoning and diseases of the circulatory and respiratory systems were higher among men, and the proportion of all hospitalization due to cancer, mental disorders, muskuloskeletal disorders and diseases of the genitourinary system were higher among women.

**Figure 4:**
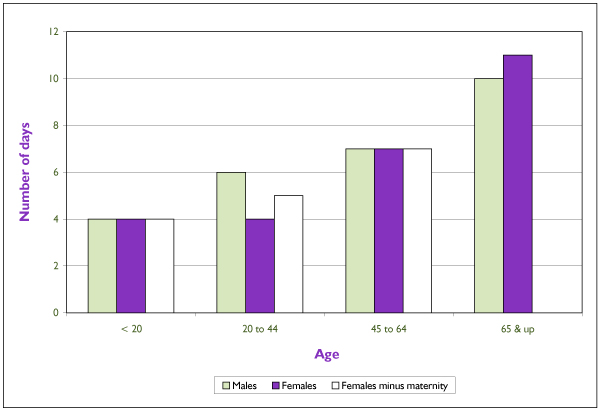
**Average Length of Stay in Acute Care Hospitals 2000–2001, Canada **Source: Hospital Morbidity Database, CIHI

It is only at older ages that women tend to stay in the hospital longer, on average, than men. As indicated in Figure 4, the average length of stay is similar between men and women aged less than 20 and those aged 45 to 64. Between the ages of 20 and 44, however, men tend to stay in the hospital longer than women of the same age, even when hospitalizations for pregnancy and childbirth are excluded.

**Figure 5:**
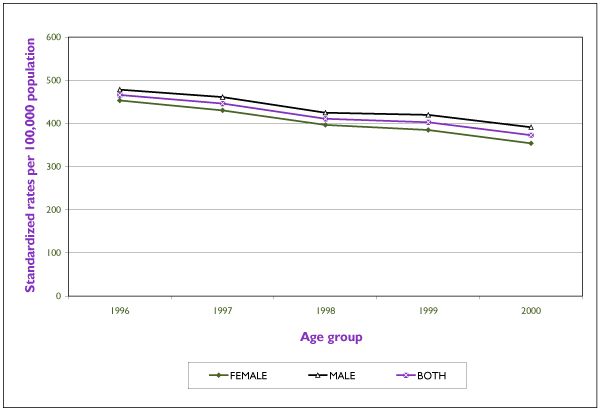
**Standardized Rates of Hospital Admissions for Ambulatory Care Sensitive Conditions by Sex, Canada **Source: Hospital Morbidity Database, CIHI

Figure 5 shows that the age-adjusted rate of hospital admissions for ambulatory-sensitive conditions have been somewhat higher among men than women over the period examined (354 per 100,000 versus 391 per 100,000 for women and men respectively in 1999). Hospitalization rates for these conditions were also lower among women in most provinces (data not shown). While comparing these rates between subgroups of the population can be useful, it must be noted here that changes over time in the rates of hospitalization for these conditions are more difficult to interpret, since they could reflect more aggressive management of ambulatory-sensitive conditions in emergency departments or better management by primary care physicians.

### Chronic Conditions and Disability

**Figure 6:**
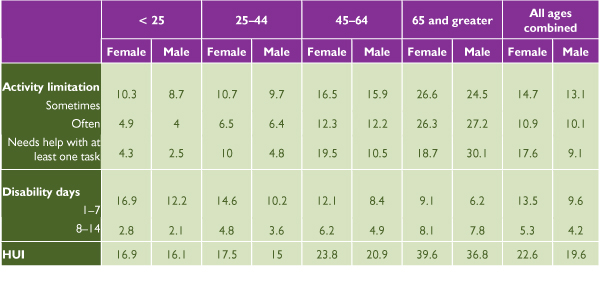
**Comparison of Disability Prevalence by Sex and Age, Using Four Definitions of Disability, Canada **Source: Canadian Community Health Survey, Statistics Canada, 2000–2001

As indicated in Figure 6, the prevalence of disability varies by age, sex and the definition used. According to the activity limitation definition, 25.6% of women and 23.2% of men report an activity limitation. However, a large portion of these report having activity limitation "sometimes," and 10.9% of women and 10.1% of men report having activity limitation "often." As expected, the HUI classifies a slightly different proportion of individuals as reporting disability (22.6%, 95% confidence interval [CI] 22.2, 23.1 versus 19.6%, 95% CI 19.1, 20.1 among women and men respectively).

**Figure 7:**
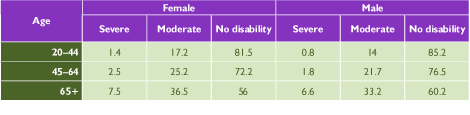
**Distribution of Disability Intensity by Age and Sex, Canada **Source: Canadian Community Health Survey, Statistics Canada, 2000–2001

**Figure 8:**
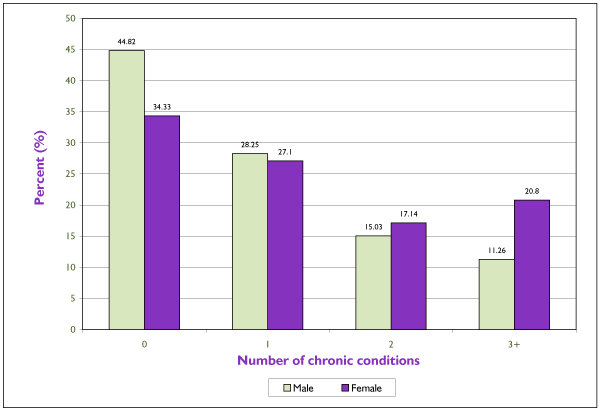
**Distribution of Chronic Conditions by Sex among those Less than 65 Years, 2001, Canada **Source: Canadian Community Health Survey, Statistics Canada, 2000–2001

**Figure 9:**
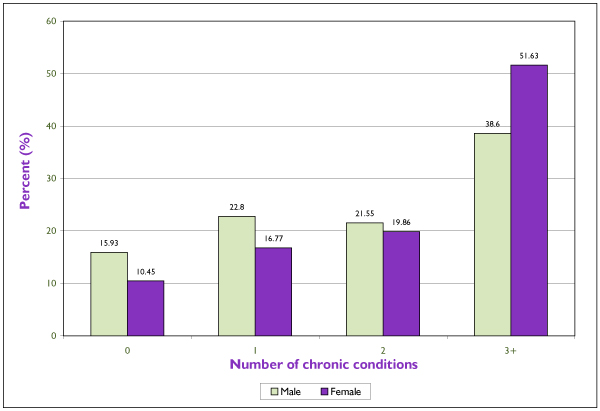
**Distribution of Chronic Conditions by sex Among Those 65 Years and Older, 2001, Canada **Source: Canadian Community Health Survey, Statistics Canada, 2000–2001

**Figure 10:**
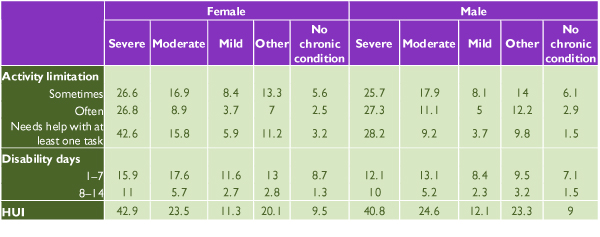
**Disability Prevalence, Using Four Definitions, by Sex and Type of Chronic Condition, Canada **Source: Canadian Community Health Survey, Statistics Canada, 2000

**Figure 11:**
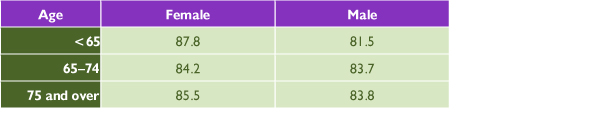
**Disability Prevalence of Disability across Age, by Sex among those in Institutions Source: National Population Health Survey Institution survey, Statistics Canada, 1996–1997. **Source: Canadian Community Health Survey, Statistics Canada, 2000

Severe as well as moderate disability was more common among women than men across all age groups (Figure 7). As expected, chronic conditions were more common among women. Interestingly, among those under 65 years of age, this sex gap was more pronounced for comorbidity (two or more reported conditions), which was significantly more common among women (Figures 8 and 9), whereas the prevalence of reporting one chronic condition only was not more common among women. As indicated in Figure 10, the prevalence of disability was higher among women than men with moderate and severe chronic conditions, and even among respondents who did not report any chronic conditions (although not statistically significant). Using all measures of disability, prevalence was highest among Aboriginal people, for both sexes, and women from all ethnic categories had a higher prevalence of disability than men (data not shown). Figure 11 shows data from the institutional component of the NPHS, indicating that most Canadian women and men living in long-term care facilities (over 80%) report disability and that a slightly larger proportion of women living in long-term care facilities report a disability as compared with men. However, there are more women than men living in long-term care facilities (data not shown).

**Figure 12:**
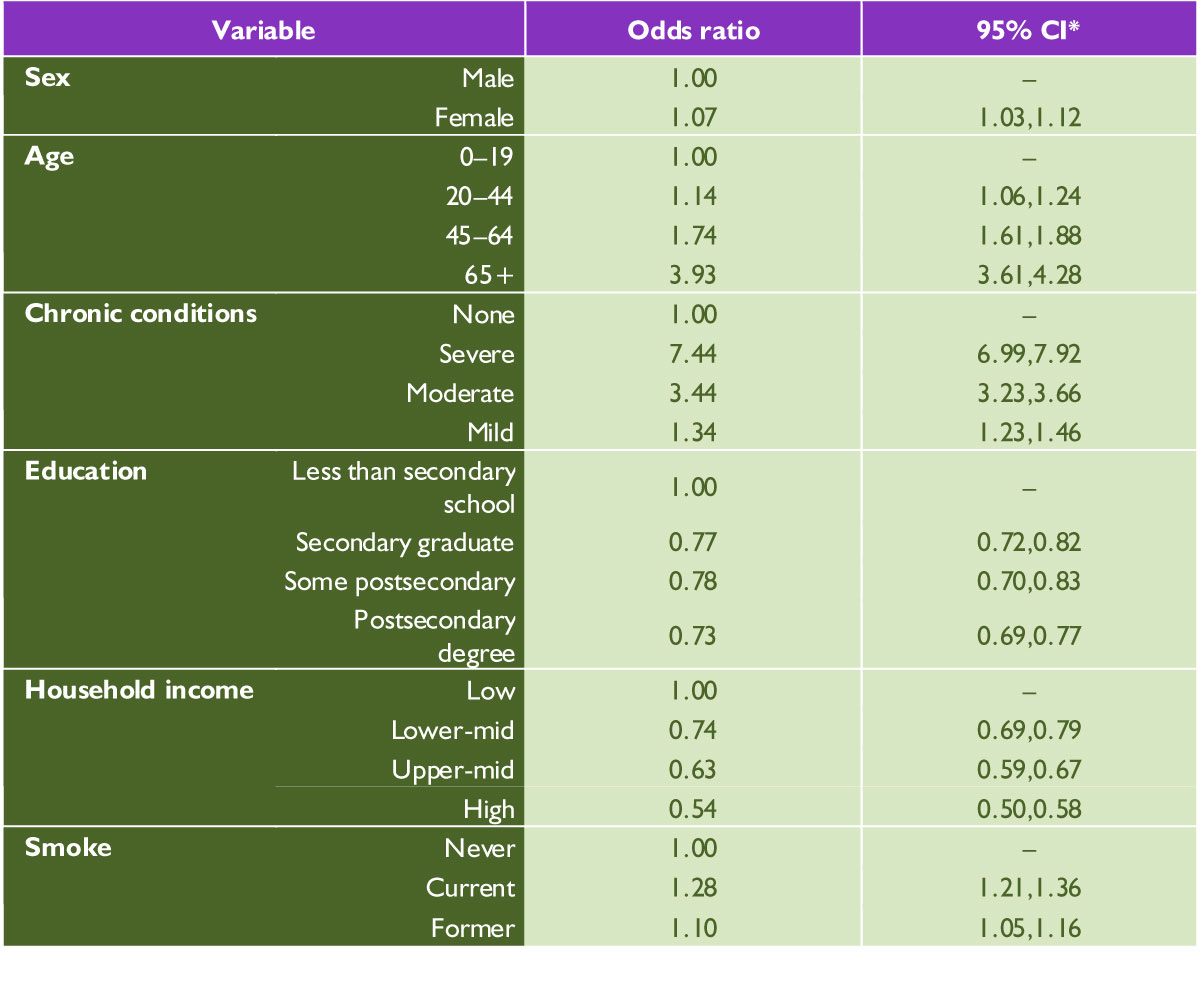
**Adjusted Odds Ratios for Disability Based on Logistic Regression Modelling **Source: Canadian Community Health Survey, Statistics Canada, 2000–2001 *Calculated using the Bootstrap method.

The results of multivariate logistic regression analysis (Figure 12) indicated that with adjustment for the effect of chronic conditions, income, education, smoking status and age, sex was only slightly associated with the prevalence of reported disability (adjusted odds ratio [OR] of 1.07, p < 0.05). All other variables were strongly associated with disability.

**Figure 13:**
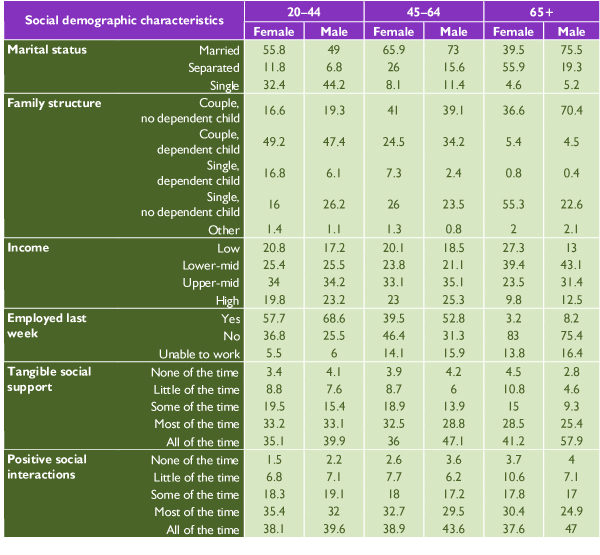
**Social Demographic Characteristics (Percentage) by Sex and Age among Those Who Have Disability (Defined Using HUI), Canada **Source: Canadian Community Health Survey, Statistics Canada, 2000–2001

Important differences in the social and demographic characteristics of disabled Canadian women as compared with men were observed (Figure 13). In particular, disabled women aged 45 and over were less likely than disabled men to be married (for example, using HUI as the definition of disability, 65.8%, 95% CI 63.7, 67.9 and 73.0%, 95% CI 71.0, 75.0 of women and men respondents were married in the 45 to 64 age group). In addition, women who reported a disability were more likely to be single with dependent children (16.8%, 95% CI 15.2, 18.3 versus 6.1%, 95% CI 4.9, 7.2 among women and men aged 20 to 44). Income and employment were also lower among disabled women than men (Figure 13). For example, 27.3% (95% CI 25.3, 29.2) of elderly women who reported a disability were in the low-income category, as compared with 13% (95% CI 11.5, 14.5) of men. Among women aged 20 to 44 who reported a disability (using the HUI), 57.7% (95% CI 55.7, 59.8) were employed the previous week, as compared with 68.9% (95% CI 66.2, 70.9) of men who reported a disability in the same age group. Women who reported a disability also had less tangible social support and positive social interactions than men with a disability, across all age groups (although the sex gap in social support was even more pronounced in older age groups). As indicated in Figure 13, 15.3% (95% CI 13.2, 17.4) of women aged over 65 who reported a disability had little or no tangible social support, as compared with 7.4% (95% CI 6.0, 8.9) of men in the same age group. The disability findings for the social and demographic characteristics were similar for all definitions of disability examined.

## Discussion

### Data Gaps and Recommendations

This chapter highlights the many disadvantages women face with respect to longer-term morbidity and disability. In particular, women have a higher prevalence of multiple chronic conditions, and severe and moderate disability. Women who report disabilities are also more likely to be poor and unemployed, and to have little or no social support as compared with men.

The overall lower rate of hospital morbidity among women (with the exception of the 20 to 44 age group) than men indicates that women's morbidity is less acute in nature, and may reflect the increased use of ambulatory care among women for conditions whose early detection and treatment and/or enhanced adherence to recommended treatment can avoid problems later on (as indicated by the analysis of hospitalizations for ambulatory care-sensitive conditions). This type of analysis would be enhanced by further examining hospital data with respect to level of urgency of care, and the proportion of hospitalizations due to elective procedures among women. Determinants of hospital morbidity, especially in younger adult women (age 20 to 44), should be examined more comprehensively. The attempt at identifying "normal" deliveries indicated that a small proportion of deliveries in hospital were completely without complications (and therefore not a morbid event). However, more work needs to be done to identify valid and reliable measures of normal deliveries, using all diagnostic codes (primary and secondary) as well as procedure codes.

As provincial and national morbidity databases are being further developed, such as those for chronic and long-term care, for rehabilitation services and for home care, studies of sex and gender differences in morbidity and uses of services will be greatly enhanced. To better understand gender differences in the use of less urgent care, such as elective procedures, information is needed on severity at referral, waiting times, and inpatient as well as same-day procedure hospital services. Person-oriented hospital morbidity data, as well as long-term longitudinal data on disability, would be useful for further exploring the patterns of transition between morbid and healthy states among women as compared with men.

The impact of disability on women's health is significant. The respective contributions of biology, disease severity and social factors on the risk of disability among women as compared with men needs further study. Longitudinal analyses examining social factors before disability (chronic conditions, socio-economic status, access to care, etc.) and after disability (change in work status, income, social interactions, etc.) would be useful to identify possible interventions for the prevention of disability, and to improve quality of life, change in work status and social interactions among women with disabilities. The study of disability among more vulnerable women, such as the elderly and women living in poverty, would be useful in order that the results might inform the development of more targeted policies and interventions. Disability affects women's lives in various ways, as women are often caregivers for children, spouses or other family members with disabilities. Tools to monitor the impact on their health and well-being need to be further developed. More work in developing new measures of disability would enhance our understanding of the magnitude of the problem among women. Measures of disability (such as the HUI) used in current health surveys have limitations for use in gender-sensitive analyses and are mainly based on physical and sensory functioning (such as walking or hearing). Other factors that may contribute significantly to disability among women (such as depression or severe fatigue) should be further explored.

## Note

The views expressed in this report do not necessarily represent the views of the Canadian Population Health Initiative, the Canadian Institute for Health Information or Health Canada.
